# Immunohistochemistry of Colorectal Cancer Biomarker Phosphorylation Requires Controlled Tissue Fixation

**DOI:** 10.1371/journal.pone.0113608

**Published:** 2014-11-19

**Authors:** Abbey P. Theiss, David Chafin, Daniel R. Bauer, Thomas M. Grogan, Geoffrey S. Baird

**Affiliations:** 1 Ventana Medical Systems Inc., Tucson, Arizona, United States of America; 2 Department of Laboratory Medicine, University of Washington, Seattle, Washington, United States of America; 3 Department of Pathology, University of Washington, Seattle, Washington, United States of America; Moffitt Cancer Center, United States of America

## Abstract

Phosphorylated signaling molecules are biomarkers of cancer pathophysiology and resistance to therapy, but because phosphoprotein analytes are often labile, poorly controlled clinical laboratory practices could prevent translation of research findings in this area from the bench to the bedside. We therefore compared multiple biomarker and phosphoprotein immunohistochemistry (IHC) results in 23 clinical colorectal carcinoma samples after either a novel, rapid tissue fixation protocol or a standard tissue fixation protocol employed by clinical laboratories, and we also investigated the effect of a defined post-operative “cold” ischemia period on these IHC results. We found that a one-hour cold ischemia interval, allowed by ASCO/CAP guidelines for certain cancer biomarker assays, is highly deleterious to certain phosphoprotein analytes, specifically the phosphorylated epidermal growth factor receptor (pEGFR), but shorter ischemic intervals (less than 17 minutes) facilitate preservation of phosphoproteins. Second, we found that a rapid 4-hour, two temperature, formalin fixation yielded superior staining in several cases with select markers (pEGFR, pBAD, pAKT) compared to a standard overnight room temperature fixation protocol, despite taking less time. These findings indicate that the future research and clinical utilities of phosphoprotein IHC for assessing colorectal carcinoma pathophysiology absolutely depend upon attention to preanalytical factors and rigorously controlled tissue fixation protocols.

## Introduction

In targeted therapy of colorectal cancer, there has been recent attention placed on the importance of identifying the activation states of cell signaling molecules to the prediction of responses to therapy. A case in point is the EGFR-mediated reactivation of MAPK signaling, which contributes to the insensitivity of BRAF-mutant colorectal carcinomas to RAF inhibition with Vemurafenib [1]. Importantly this pEGFR reactivation event is subject to modulation by EGFR inhibitors, and the combined polytherapy BRAF and EGFR inhibitors overcome this resistance, with beneficial response *in vitro* and in animal models *in vivo*.

The extension of these prior laboratory findings to the clinic, and our ability to affect alternative treatments, is entirely dependent on our ability to accurately preserve and detect the activation states in question. In seeking to determine the optimum preservation (tissue fixation) conditions to support targeted therapy, the literature has led us to question whether or not standard practice in the anatomic pathology laboratory is sufficient to address all of the potential pitfalls that could effect biomarker/activation state preservation. For example, in our own clinical laboratory experience, tissue samples can remain at room temperature prior to immersion in fixative for lengthy periods, and the actual time spent in a fixative, and the temperature of that fixative, are sometimes poorly controlled. Current US professional guidelines on this issue [2]–[4] address only a limited number of clinical sample types and downstream biomarker analyses, or are still quite broad in defining what is considered acceptable in terms of fixation time (a 2-fold variation in maximum fixation time, 16–32 hours, is recommended in CLSI guidelines, and a 12-fold range of fixation duration, 6–72 hours, is considered acceptable in ASCO/CAP breast cancer marker guidelines). A pre-fixation “cold ischemia” time of up to one hour is also deemed acceptable in one of these guidelines [3], which is within the range of what we have observed clinically in our own institutions. Previous cancer biomarker immunohistochemistry studies have indeed found ∼1 hour ischemia times acceptable, although these studies have focused primarily on established biomarkers such as Estrogen Receptor and HER2 [5], [6]. However, of the guidelines that do exist, none purport to address the preservation of biomarker activation states, such as phosphorylation states, at all. This is particularly problematic, because phosphorylation states have been known for some time to be highly sensitive to preanalytical conditions [7]–[9], including warm ischemic times [10] (which are not investigated here).

To this end, we developed a novel, rapid two-temperature formalin fixation strategy that we found preserves key features of tissue such as morphology, as well as protein expression as assessed by immunohistochemistry [11]. In that work, we found significant pAKT preservation in a Calu3 mouse xenograft model, as assessed by IHC, using a two-temperature fixation protocol, and we found that this staining was almost entirely lost using standard room temperature fixation. Moving forward from this study, we extended our study into colorectal carcinoma samples, analyzing for additional phosphoepitopes. In this current comprehensive study, we asked whether or not the two-temperature technology additionally aided in preserving the activation state of biomarkers relevant to colorectal cancer therapy, as well as whether or not controlling pre-fixation cold ischemic intervals was important for these biomarkers. Using 23 colorectal carcinoma surgical samples collected with defined cold ischemic intervals and formalin fixation protocols, we studied the phosphorylation state of numerous PI3 Kinase pathway targets, as well as the BRAF V600E mutation and other markers using immunohistochemistry [12], [13]. We report here on both the importance of cold ischemic time as a variable in phosphoprotein analysis, as well as the overall impact of our rapid two-temperature formalin fixation protocol.

## Materials and Methods

### Sample Collection and Fixation

Human colon carcinoma samples were obtained from Indivumed GmBH (Hamburg, Germany) as paraffin-embedded blocks. Samples were collected at Indivumed because Ventana is not a medical facility and thus has no access to surgical samples, and also because Indivumed was known to be able to provide tissue samples with a documented short (less than 17 minutes) post-excision ischemic interval. Indivumed scientists performed tissue collections under approval from their local institutional review board, and obtained written consent from the patients involved.

For all experiments, fixation protocols were carried out by Indivumed scientists, and consisted of immersion in 10% neutral buffered formalin (10% saturated aqueous formaldehyde from Fisher Scientific, Houston, TX, buffered to pH 6.8–7.2 with 100 mM phosphate buffer) for varying times at temperatures ranging from 4–45°C. Room temperature ranged from 20–25°C. Tissue samples collected from clinical cases were split into thirds and each fixed differently. One piece was wrapped in saline soaked gauze and kept at room temperature for 1 hour (“1 hour cold ischemia”) before 24 hr fixation at RT. This was to test the effect of the longer guideline-prescribed cold ischemia interval of one hour. The remaining two pieces were to test the effect of shorter (less than 17 minutes) cold ischemia interval. One piece was placed directly into room temperature formalin for 24 hours (“24 hr”). A third piece was placed directly into 4°C formalin for 2 hours followed by 2 hours in 45°C formalin (“2+2”) [11]. The latter was to test if the process could be sped up to increase work efficiency in the hospital. Fixation was carried out in 100–500 mL covered beakers in a refrigerator for 4°C treatment, or in a standard chemical fume hood for higher temperatures. Cold ischemia times for the “24” and “2+2” samples averaged 10+/−3 minutes (range 6–17 min). Downstream tissue processing was performed as described [11], with all solvent processor steps kept at 45°C under ambient pressure, and the paraffin step was held at 60°C under vacuum. All specimens were embedded in paraffin and cut onto glass slides as 4-micron sections for IHC or histomorphology.

### Automated Immunohistochemistry

Immunohistochemistry assays were performed on a VENTANA Discovery XT automated staining instrument according to the manufacturer's instructions. Slides were de-paraffinized using EZprep solution (Ventana Medical Systems, Inc., Tucson, AZ) for 30 minutes at 75°C. Epitope retrieval was accomplished on the automated stainer with CC1 solution (Ventana Medical Systems, Inc., Tucson, AZ) for 64 minutes at 95°C. Antibodies obtained from Cell Signalling Technologies or Epitomics were first titered over a range of concentrations to provide the optimum ratio of specific staining to background staining. Once titers were set, antibodies were transferred with diluent to user fillable dispensers for use on the automated stainer. Slides were developed using the Opti*view* DAB detection kit (Ventana Medical Systems, Inc., Tucson, AZ). Briefly, steps included inhibitor for 8 minutes, linker for 8 minutes, multimer for 12 minutes, DAB/peroxide for 8 minutes and copper for 4 minutes. Slides were then counterstained with hematoxylin II for 8 minutes (Ventana Medical Systems, Inc., Tucson, AZ). Antibodies, clones, and titers are listed in Table 1. Antibody titers were determined for each antibody using positive and negative control tissues following the manufacturer's instructions.

**Table 1:** All antibodies used for detection in this study.

Antibody Target	Clone	Dilution	Phosphorylation Site, if applicable	Vendor	Cat #
Phospho-EGFR	EP774Y	1∶2400	Tyr1068	Epitomics, Burlingame, CA	1727-1
Phospho-MSK1	Polyclonal	1∶50	Thr581	Cell Signalling Technologies, Danvers, MA	9595
EGFR	3C6	Predilute		Ventana Medical Systems, Inc, Tucson, AZ	760-2988
PTEN	D4.3	1∶80		Cell Signalling Technologies, Danvers, MA	9188
BRAF-V600E	VE1	Predilute		Ventana Medical Systems, Inc, Tucson, AZ	790-4855
Phospho-MEK1/2	166F8	1∶50	Ser221	Cell Signalling Technologies, Danvers, MA	2338
Phospho-MTOR	49F9	1∶200	Ser2448	Cell Signalling Technologies, Danvers, MA	2976
Phospho-AKT	D9E	1∶50	Ser473	Cell Signalling Technologies, Danvers, MA	4060
Phospho-ERK	20G11	1∶400	Thr202/Tyr204	Cell Signalling Technologies, Danvers, MA	4376
Phospho-PRAS40	C77D7	1∶250	Thr246	Cell Signalling Technologies, Danvers, MA	2997
Phospho-BAD	40A9	1∶40	Ser112	Cell Signalling Technologies, Danvers, MA	5284

### Immunohistochemistry Analysis

Immunohistochemical analyses were scored by two independent pathologists, each blinded to the fixation methodology for each slide, using an H-score for semiquantitation of staining proportion and intensity [14]. In cases with both *in situ* and invasive neoplasia, only the invasive tumor away from the margins of the tissue sample was scored. High intensity staining that was clearly relegated to the absolute tissue margins (i.e. what is commonly referred to in clinical immunohistochemistry as an “edge effect” [15]) was ignored. Scores were unblinded by a third party, and all H scores from each pathologist were plotted against each other for each marker. Scores that appeared subjectively discrepant, i.e. those that substantially deviated from the line of identity as assessed by visual inspection, were reviewed jointly on a multi-headed microscope and a consensus score was generated.

### Statistical Analysis

**Figure 1:**
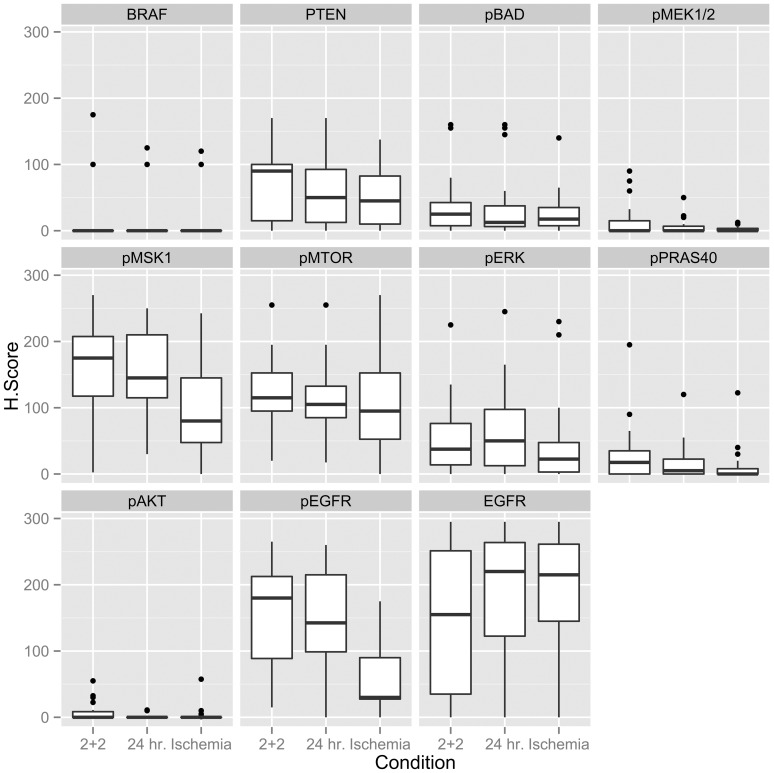
Phosphoprotein IHC scoring. H scores of staining intensity for all studied biomarkers, by treatment condition. “2+2” is the rapid fixation condition, “24 hr.” is the standard 24-hour room temperature condition without pre-fixation cold ischemia, and “Ischemia” denotes a 1-hour cold ischemic period prior to 24-hour room temperature fixation. The medians are represented by horizontal lines, the boxes in plots correspond to the 25^th^ and 75 percentiles of each distribution, the whiskers extend to the most extreme value that is within 1.5 times the distance between the first and third quartiles, and data beyond the whiskers are shown as dots.

All analysis was performed using the R statistics package. For each case, the H scores from the two reviewers were averaged. Statistical differences between fixation groups (24 hr vs 2+2, 24 hr vs cold ischemia) using a two-sided paired Wilcoxon Signed Rank test. Because multiple conditions were assessed simultaneously, p-values for these comparisons were converted into false discovery rate-corrected q-values [16], [17]. Boxplots in Figure 1 were generated with the package ggplot2. Black bars represent median values and boxes extend from the 25^th^ to 75^th^ percentiles. Whiskers extend to 1.5× the interquartile distance.

## Results

H-scores for all markers are graphically represented in boxplots Figure 1, and the results of comparison testing are shown in Table 2.

**Table 2:** Comparison of Pathologist H-scoring between conditions for all tested biomarkers.

	Median Pathologist H-Score			2+2 vs. 24 hr Paired Wilcoxon Signed Rank Test		Short vs. Long: 2+2 vs. 1 hr Ischemia Paired Wilcoxon Signed Rank Test	
IHC Biomarker	2+2	24 hr	1 hr Ischemia	p value	q value	p value	q value
BRAF V600E	0	0	0	1	0.37	1	0.79
PTEN	90	50	45	0.017	0.08	0.41	0.65
pBAD	25	12.5	17.5	0.19	0.12	0.76	0.79
pMEK1/2	0	0	0	0.15	0.11	0.18	0.34
pMSK1	175	145	80	0.46	0.23	3.50E-05	3.30E-04
pMTOR	115	105	95	0.11	0.1	0.65	0.77
pERK	37.5	50	22.5	0.52	0.23	0.04	0.13
pPRAS40	17.5	5	0	0.1	0.1	0.12	0.28
pAKT	0	0	0	0.07	0.1	0.61	0.77
pEGFR	180	142.5	30	0.73	0.29	9.90E-05	4.70E-04
EGFR	155	220	215	0.1	0.1	0.84	0.79

**Figure 2:**
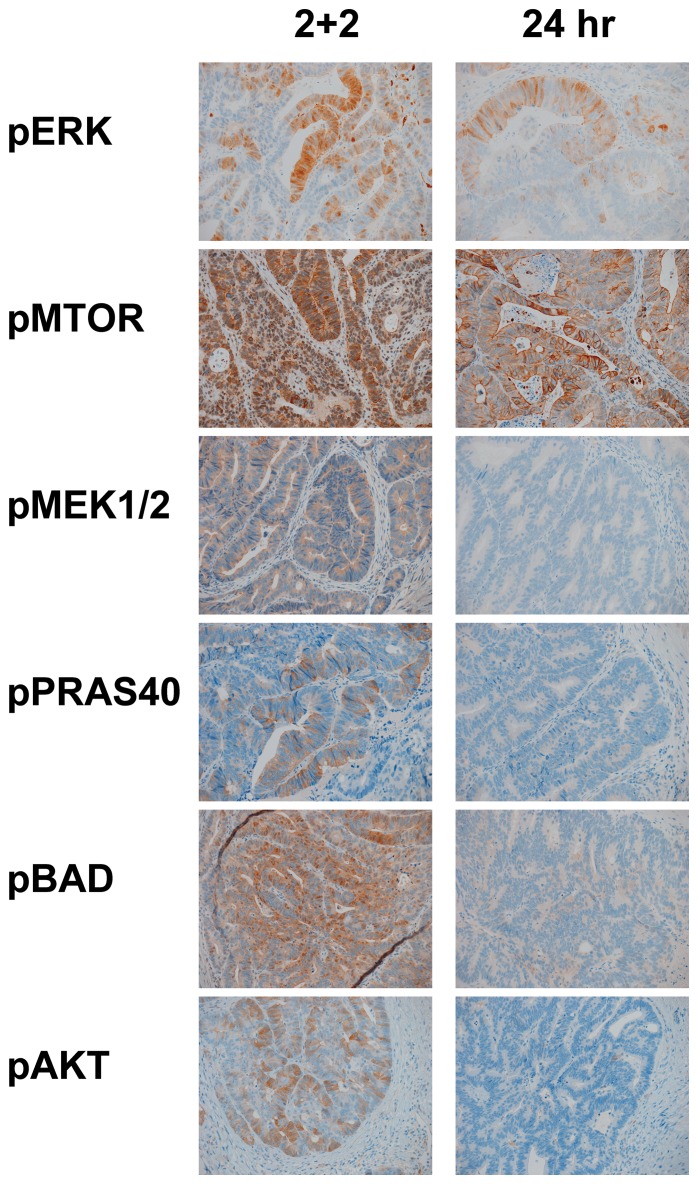
Colon Cancer Phosphoprotein IHC. Representative results from IHC analysis of two colonic carcinoma samples subjected to either the rapid fixation condition (“2+2”) or standard 24-hour room temperature condition without pre-fixation cold ischemia (“24 hr”). pERK, pMTOR, pMEK1/2 and pPRAS40 are shown for one case and pBAD and pAKT are shown from a second case. Two different cases are shown here because not all cases showed staining for all markers. All micrographs are taken at 200× with equivalent exposures.

Very few cases showed any appreciable staining for three of the markers (BRAF V600E, pMEK1/2, and pAKT), but two cases were unambiguously positive for the BRAF V600E mutation, a finding confirmed by molecular analysis (data not shown). Overall, the 2+2 protocol yielded as much or more IHC signal than the 24 hr treatment, with representative staining results shown in Figure 2. While several case-specific differences are highlighted in Figure 2, there were no statistically significant differences observed on multiple paired comparisons across the 24 cases (Table 2). Table 2 demonstrates that two markers appeared to have slightly increased staining in the 24 hr treatment (EGFR and pERK), but again, these differences were not statistically significant, nor did the pathologists scoring the samples find any case where discrepant staining between 2+2 and 24 hour conditions would lead to a change in pathophysiologic interpretation of these two markers.

**Figure 3:**
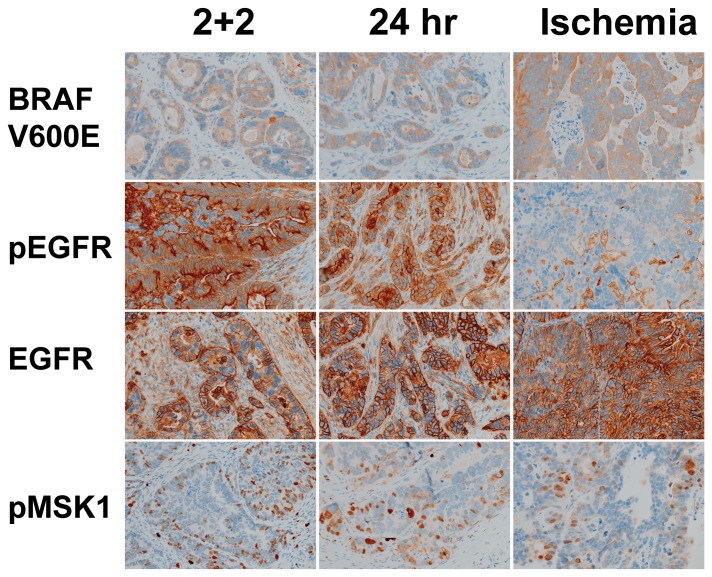
Colon Cancer Phosphoprotein IHC. Representative images of one colonic carcinoma case stained with antibodies to BRAF V600E, pEGFR, EGFR, and pMSK1, showing significant loss of pEGFR staining in the ischemia condition. All micrographs are taken at 200× with equivalent exposures.

**Figure 4:**
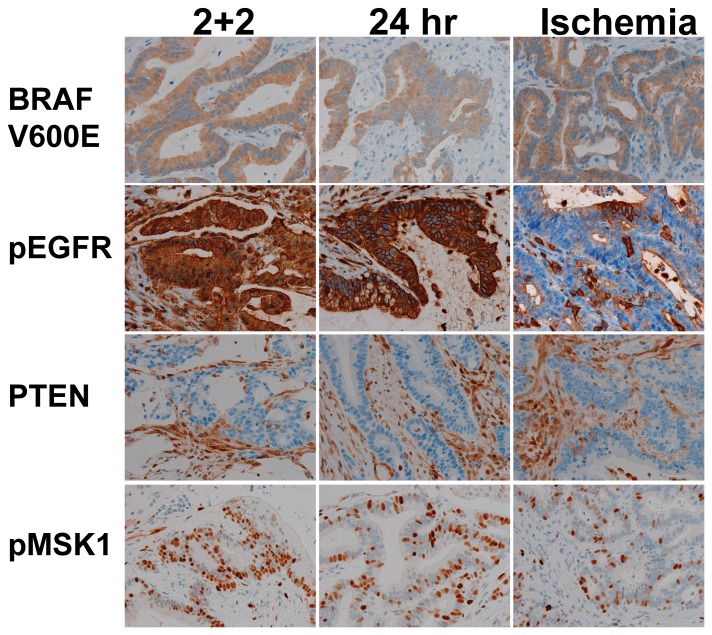
Colon Cancer Phosphoprotein IHC. Representative of a second colonic carcinoma case stained with antibodies to BRAF V600E, pEGFR, PTEN, and pMSK1, showing significant loss of pEGFR staining in the ischemia condition. All micrographs are taken at 200× with equivalent exposures.

The IHC signal was much more deleteriously affected by the longer 1-hour cold ischemia period prior to fixation when compared to the shorter ischemic interval protocols, with the sole exception of EGFR. These trends were highly statistically significant for pEGFR and pMSK1, and the reduction in pERK staining approached statistical significance. In two cases in specific, shown in Figures 3 and 4, carcinomas harboring the BRAF V600E mutation were found to be clearly positive for pEGFR in both of the shorter ischemic interval conditions, but staining was almost entirely absent when the sample was exposed to the longer 1 hour cold ischemia condition.

## Discussion

The findings of this study clearly demonstrate that a substantial post-surgical, pre-fixation cold ischemic interval (one hour) confounds the attempt to identify the phosphorylation states of a key PI3 Kinase pathway element in formalin-fixed tissue. This finding is consistent with previous reports that indicate the significant preanalytical sensitivity of phosphoprotein assays [7], [18]. If careful specimen handling is employed to minimize the cold ischemic interval (less than 17 minutes), however, both 24-hour room temperature formalin fixation and our rapid two-temperature fixation protocol preserve phosphorylation signals on average for essentially all markers investigated. Why some markers were more affected by cold ischemia is unclear to us; the balance between unhindered kinase and phosphatase activity during cold ischemic intervals is not likely to play out identically for each phosphorylation target, however, so identifying those targets with exaggerated sensitivities to cold ischemia through studies such as this one will be vitally important as the field of phosphoprotein immunohistochemistry advances.

When prefixation cold ischemia time extends to the one hour deemed an acceptable interval in the current ASCO/CAP guidelines for ER testing [3], we found that the signal from selected phosphoproteins diminishes significantly. Specifically, in two specimens found to harbor the BRAF V600E mutation (Figures 3 and 4), the 1-hour cold ischemic interval appeared to obscure the expression of pEGFR, an error that could lead to the failure to identify these patients as candidates for additional therapy [12], [13], [19]. In these two cases, the rapid two-temperature fixation protocol yielded visibly more pEGFR staining than the 24-hour fixation protocol, indicating that the rapid protocol could be a more robust methodology for assessing this clinically significant biomarker activation state. A similar trend is observed in some additional markers (eg. pBAD, pAKT) in the selected cases shown in Figure 2. This finding clearly accentuates the potential impact of preanalytical specimen handling in the era of personalized medicine, since the preservation of biomarker activation states appears to be relatively constant, on average, between fixation protocols for some biomarkers, but differs substantially for specific cases.

Several studies have now identified that MAPK pathway reactivation constitutes a mechanism of therapy resistance in BRAF V600E mutated colorectal carcinomas that are treated with Vemurafenib [1], [20], [21]. *In vitro* and xenograft models indicate that this resistant state could be overcome by dual therapy including EGFR inhibitors directed at pEGFR, but this approach is predicated on the ability of diagnostician to identify pEGFR overexpression in the clinical tissue sample. While the existing literature indicates that dual therapy holds much potential benefit for the patients with these specific tumor phenotypes, our findings indicate that all of this potential benefit is at risk if an uncontrolled tissue preservation and fixation process is employed. The two patients whose carcinomas harbored BRAF V600E mutations, for example, would not be identified as candidates for EGFR-directed therapy had their samples been collected with the longer one hour cold ischemic interval that is not only seen in clinical pathology areas, but is also deemed acceptable in a current cancer biomarker analysis guideline. Beyond these clinical considerations, it is also worth considering the extreme costs associated with novel targeted therapies, such that misidentifying patients as candidates for therapies could be not only personally harmful but also economically wasteful.

Besides the BRAFV600E/pEGFR example, it is also notable that pERK expression is decreased after 1 hour cold ischemia (see Table 2). Since pERK has been used to monitor therapy efficacy, this effect, if present in a clinical assay, could potentially impair therapy monitoring. Also, in selected cases (Figure 2), apparent pBAD expression may be reduced by prefixation ischemia, confounding our ability to assess the expression of a factor known to be relevant to colon cancer pathogenesis [19].

One potential weakness of this study is that relatively few samples were studied. While all samples were analyzed for all markers, the high cost and difficulty of obtaining samples with precisely defined preanalytical treatments from our commercial vendor precluded the study of more samples. Clearly, additional larger studies would be beneficial to confirm these findings. Work on xenograft models might also be expected to be beneficial in this area, and it has indeed been so in our prior work on this topic [11] because of the exquisite control over preanalytical conditions that is possible in those systems. However, we chose to work on clinical samples here because of their increased relevance to current problems in translational cancer research. It should be noted that the 10-minute average cold ischemia times of samples collected under this research protocol are not typically observed in current anatomic pathology practices, nor may such fast cold ischemia times be achievable with current processes in place in clinical laboratories. While it may be a challenge to incorporate our findings into standard clinical workflows because many current medical facilities are not able to reduce ischemic times significantly, we believe our data indicates that standardization and optimization of preanalytical conditions is at least a worthy goal.

Another potential weakness is that phosphoprotein signal scores across treatment conditions were not compared using clinically validated score thresholds, simply because no such thresholds exist for the markers investigated here. What we observed with our semiquantitative approach, however, was that signals were often dramatically reduced in samples with a long prefixation ischemic interval, and that in the two specific cases we discussed, the long prefixation ischemic interval appeared to ablate all relevant signal from a critical biomarker. In these cases, the controlled fixation protocols we investigated preserved signals that would likely be interpreted, in the medical judgment of two practicing pathologists, as pathophysiologically relevant to potential therapy.

Finally, while we have interpreted losses of phosphoprotein IHC signals as indications of deleterious preanalytical effects, it could be the case that increases in staining were the true artifacts and that decreased staining was closer to the true state *in situ*. However, others have demonstrated that numerous phosphomarkers, including pAKT, are clearly diminished in the context of altered fixation conditions or cold ischemia [22], [23], so our interpretation is not without precedent. While this question may never be resolved without future *in vivo* studies, what is absolutely clear from this work is that changes in the apparent activation states of many relevant cancer biomarkers appear to be sensitive to common preanalytical variables. Of these variables, the ischemic interval is perhaps one of the most important, and therefore we believe that further research and clinical studies must incorporate controls that address this factor.

## Supporting Information

Table S1 **Raw H-score data for all cases examined in this study.**
(XLSX)
